# Serial deletion reveals structural basis and stability for the core enzyme activity of human glutaminase 1 isoforms: relevance to excitotoxic neurodegeneration

**DOI:** 10.1186/s40035-017-0080-x

**Published:** 2017-04-20

**Authors:** Yuju Li, Justin Peer, Runze Zhao, Yinghua Xu, Beiqing Wu, Yi Wang, Changhai Tian, Yunlong Huang, Jialin Zheng

**Affiliations:** 1 Center for Translational Neurodegeneration and Regenerative Therapy, Shanghai Tenth People’s Hospital affiliated to Tongji University School of Medicine, Shanghai, China; 2 Department of Pharmacology and Experimental Neuroscience, University of Nebraska Medical Center, Omaha, NE USA; 3 Department of Pathology and Microbiology, University of Nebraska Medical Center, Omaha, NE USA; 4 Shanghai Tenth People’s Hospital affiliated with Tongji University School of Medicine, Shanghai, 200072 China; 5 Laboratory of Neuroimmunology and Regenerative Therapy, Departments of Pharmacology and Experimental Neuroscience and Pathology and Microbiology, 985930 Nebraska Medical Center, Omaha, NE 68198-5930 USA

**Keywords:** Glutaminase 1, Protein expression, Enzyme activity

## Abstract

**Background:**

Glutaminase 1 is a phosphate-activated metabolic enzyme that catalyzes the first step of glutaminolysis, which converts glutamine into glutamate. Glutamate is the major neurotransmitter of excitatory synapses, executing important physiological functions in the central nervous system. There are two isoforms of glutaminase 1, KGA and GAC, both of which are generated through alternative splicing from the same gene. KGA and GAC both transcribe 1–14 exons in the N-terminal, but each has its unique C-terminal in the coding sequence. We have previously identified that KGA and GAC are differentially regulated during inflammatory stimulation and HIV infection. Furthermore, glutaminase 1 has been linked to brain diseases such as amyotrophic lateral sclerosis, Alzheimer’s disease, and hepatic encephalopathy. Core enzyme structure of KGA and GAC has been published recently. However, how other coding sequences affect their functional enzyme activity remains unclear.

**Methods:**

We cloned and performed serial deletions of human full-length KGA and GAC from the N-terminal and the C-terminal at an interval of approximately 100 amino acids (AAs). Prokaryotic expressions of the mutant glutaminase 1 protein and a glutaminase enzyme activity assay were used to determine if KGA and GAC have similar efficiency and efficacy to convert glutamine into glutamate.

**Results:**

When 110 AAs or 218 AAs were deleted from the N-terminal or when the unique portions of KGA and GAC that are beyond the 550 AA were deleted from the C-terminal, KGA and GAC retained enzyme activity comparable to the full length proteins. In contrast, deletion of 310 AAs or more from N-terminal or deletion of 450 AAs or more from C-terminal resulted in complete loss of enzyme activity for KGA/GAC. Consistently, when both N- and C-terminal of the KGA and GAC were removed, creating a truncated protein that expressed the central 219 AA - 550 AA, the protein retained enzyme activity. Furthermore, expression of the core 219 AA - 550 AA coding sequence in cells increased extracellular glutamate concentrations to levels comparable to those of full-length KGA and GAC expressions, suggesting that the core enzyme activity of the protein lies within the central 219 AA - 550 AA. Full-length KGA and GAC retained enzyme activities when kept at 4 °C. In contrast, 219 AA - 550 AA truncated protein lost glutaminase activities more readily compared with full-length KGA and GAC, suggesting that the N-terminal and C-terminal coding regions are required for the stability KGA and GAC.

**Conclusions:**

Glutaminase isoforms KGA and GAC have similar efficacy to catalyze the conversion of glutamine to glutamate. The core enzyme activity of glutaminase 1 protein is within the central 219 AA - 550 AA. The N-terminal and C-terminal coding regions of KGA and GAC help maintain the long-term activities of the enzymes.

## Background

Glutaminase is an important metabolic enzyme that catalyzes the deamidation of glutamine to glutamate. The hydrolysis occurs in the inner membrane of mitochondria, which generates stoichiometric amounts of glutamate and ammonia [1–3]. Mammalian cells typically express two kinds of glutaminase: Glutaminase 1 (GLS1, kidney-type glutaminase) and Glutaminase 2 (GLS2, liver-type glutaminase). GLS1 is highly expressed in the central nervous system (CNS) and kidney, whereas GLS2 is highly expressed in the liver. Despite similar names, GLS1 and GLS2 are encoded by different genes [4]; *GLS1* gene is located on chromosome 2, whereas *GLS2* gene is located on chromosome 12 in the human genome [5]. *GLS1* is known to have two main isoforms, kidney-type glutaminase isoform (KGA) and glutaminase C isoform (GAC), due to alternative splicing. KGA and GAC transcribe the same 14 exons in the N-terminal, but each transcribes a unique C terminal from the *GLS1* gene. *KGA* transcribes exons 16-19, resulting in a protein with 669 amino acids (AAs); while *GAC* transcribes exon 15, generating a protein with 598 AAs [6].

As a metabolic enzyme, glutaminase is known to promote cancer cell proliferation and transformation [7, 8]. Glutaminase is a crucial initial enzyme in the glutaminolysis pathway, providing an energy source for proliferating cells and nitrogen substrates for lipid and protein synthesis. During glutaminolysis, glutamine is converted by glutaminase into glutamate and subsequently into α-ketoglutarate, which then enters the TCA cycle [9, 10]. In addition, glutamate can be converted into glutathione, an anti-oxidant that helps protect cancer cells from oxidative stress and promotes chemoresistance [11, 12]. Interestingly, GAC isoform is overexpressed in many human cancers [13], while KGA was found to be decreased in lung tumors compared with normal lung tissue.

In the central nervous system, Glutaminase 1 performs important functions in neurons by providing glutamate as a key excitatory neurotransmitter. However, excess levels of glutamate over-excite neurons and lead to a form of neuronal cell death known as excitotoxicity. Excitotoxicity has been linked to the pathogenic processes of various CNS disorders [14–17] and neurodegenerative diseases including HIV-1-associated neurocognitive disorders (HAND). Interestingly, glutaminase has been linked to brain diseases such as HIV-1 associated dementia, multiple sclerosis, amyotrophic lateral sclerosis, and Alzheimer’s disease [18–22], as well as schizophrenia [23]. The exact mechanism of how glutaminase contributes to brain disorders remains unclear. However, enzymatic conversion of glutamine to glutamate presents two potential problems: improper compartmentalization of glutamate interfering with signal transduction and glutamate-induced excitotoxicity. An increase in the amount, activity or release of glutaminase could facilitate uncontrolled generation of glutamate in the CNS extracellular space. Indeed, aberrant release of glutaminase through extracellular vesicles can be found in diverse conditions [24, 25]. In addition, a genetic variant in the promoter of glutaminase is associated with hepatic encephalopathy [26, 27].

Bacterial expression systems have been used to characterize glutaminase from different species including Rat, *Bacillus licheniformis*, *cyanobacterium Synechocystis*, *cyanobacterium Anabaena*, and *Micrococcus luteus* [9, 28–31]. More recently, the crystal structures of both full-length protein and the catalytic domain of human glutaminase 1 have been published, lending important insights into the enzymes [32–35]. However, how different coding regions affect the glutaminase enzyme activities of KGA and GAC remains unclear. In the current study, we successfully cloned human KGA and GAC and subsequently expressed them through a bacterial expression system. Purified proteins of KGA and GAC showed similar efficacy to convert glutamine to glutamate. Furthermore, we used sequential deletion approach and determined the main activity domain of both KGA and GAC in a glutaminase enzyme activity assay. Surprisingly, while the 219 AA - 550 AA core enzyme region retained glutaminase enzyme activities in the short term, it lost activities more readily compared with full-length KGA and GAC, suggesting that both N-terminal and C-terminal coding regions are important for the long-term enzyme activities of KGA and GAC. Understanding the structural basis and stability of the glutaminase isoforms will help to identify key mechanisms of the degenerative process in a wide spectrum of neurological diseases.

## Methods

### Cloning

The full length coding domain sequence (CDS) of KGA and GAC were amplified from human cDNA library (PCR system: Pfu Ultra II, from Agilent). Due to GC rich sequence in the 5’ terminal, we used 5× GC buffer for the PCR reaction. The PCR cycles were set as follows: First step, 96 °C 5 min; Second step, 98 °C 20 s; Third step, 60 °C 35 s; Fourth step, 72 °C 2 min 30 s; Fifth step, repeat the second, third and fourth steps for 35 cycles; Sixth step, 72 °C 10 min. The PCR products were cloned into the vector pET-28a (+) (Addgene) using the restriction enzymes BamHI / XhoI (New England Biology). New constructs were confirmed by sequencing through an Applied Biosystems (ABI) 3730 48-capillary electrophoresis DNA analyzer at UNMC DNA Sequencing Core facility. Primers for the cloning and series deletions are shown in Table 1. The constructs pET-28a-GAC and pET-28a-KGA served as the PCR templates for generating series deletion mutants. All deletion mutants were ligated to pET-28a (+) at the site of BamHI / XhoI, and confirmed with sequencing.

**Table 1:** Primer sequences for amplifying full length KGA, full length GAC and all the deletions

KGA 1AA - 669AA	Forward: AGGATCCATGCGGCTGCGAGGCTCGGGGATGCTG
	Reverse: GCTCGAGTTACAACAATCCATCAAG
KGA 111AA - 669AA	Forward: TGGATCCATGGAGACGGACGCGTTTGGCAAC
	Reverse: GCTCGAGTTACAACAATCCATCAAG
KGA 219AA - 669AA	Forward: CGGATCCATGGTGATTCCTGACTTTATGTC
	Reverse: GCTCGAGTTACAACAATCCATCAAG
KGA 311AA - 669AA	Forward: TGGATCCATGGAGCCGAGTGGACTAAGATTC
	Reverse: GCTCGAGTTACAACAATCCATCAAG
KGA 415AA - 669AA	Forward: TGGATCCATGCAGCTGTGCTCCATTGAAGTG
	Reverse: GCTCGAGTTACAACAATCCATCAAG
GAC 1AA - 598AA	Forward: AGGATCCATGCGGCTGCGAGGCTCGGGGATGCTG
	Reverse: GCTCGAGTTAGCTTTTCTCTCCCAGAC
GAC 111AA - 598AA	Forward: CCTCGAGTCCTTCAGCAATTGTATAG
	Reverse: GCTCGAGTTAGCTTTTCTCTCCCAGAC
GAC 219AA - 598AA	Forward: CGGATCCATGGTGATTCCTGACTTTATGTC
	Reverse: GCTCGAGTTAGCTTTTCTCTCCCAGAC
GAC 311AA - 598AA	Forward: TGGATCCATGGAGCCGAGTGGACTAAGATTC
	Reverse: GCTCGAGTTAGCTTTTCTCTCCCAGAC
GAC 415AA - 598AA	Forward: TGGATCCATGCAGCTGTGCTCCATTGAAGTG
	Reverse: GCTCGAGTTAGCTTTTCTCTCCCAGAC
GLS1 1AA - 550AA	Forward: AGGATCCATGCGGCTGCGAGGCTCGGGGATGCTG
	Reverse: GCTCGAGCCTTTGATCACCACCTTCTC
GLS1 1AA - 450AA	Forward: AGGATCCATGCGGCTGCGAGGCTCGGGGATGCTG
	Reverse: GCTCGAGAGGGCTCAGTACTCTTTCACCA
GLS1 1AA - 344AA	Forward: AGGATCCATGCGGCTGCGAGGCTCGGGGATGCTG
	Reverse: GCTCGAGTAGTGAAGTCACAACAATTGC
GLS1 1AA - 247AA	Forward: AGGATCCATGCGGCTGCGAGGCTCGGGGATGCTG
	Reverse: GCTCGAGTGCAACCTTTCCTCCAGACT
GLS1 1AA - 150AA	Forward: AGGATCCATGCGGCTGCGAGGCTCGGGGATGCTG
	Reverse: CCTCGAGTCCTTCAGCAATTGTATAG
GLS1 219AA - 550AA	Forward: CGGATCCATGGTGATTCCTGACTTTATGTC
	Reverse: GCTCGAGCCTTTGATCACCACCTTCTC

### Prokaryotic expression for recombinant protein

Plasmids for KGA and GAC deletion mutants were transformed into the BL21 (DE3) strain (Invitrogen, Carlsbad, CA) individually for prokaryotic expression. Bacteria clones were cultured in the LB (Luria broth) with kanamycin in an incubator shaker at 37 °C and 250 rpm overnight (New Brunswick). The next day, 50 μL medium containing bacteria was put into 50 ml fresh LB with kanamycin and incubated again; when the OD (Optical Density) reached 0.6, IPTG (Isopropyl β-D-1- thiogalactopy- ranoside) was added to the medium at a final concentration of 0.1 mM. After 5 h induction at 37 °C and 150 rpm, bacteria were collected by centrifugation (4500 × g for 10 min). Supernatants were discarded and pellets were kept for cell lysis and protein purification. Briefly, pellets were put in cold buffer (50 mM NaH2PO4 · H2O, 500 mM NaCl, 10 mM Imidazole, pH 8.0) for sonication to release the recombinant proteins. The lysates were centrifuged again at 10,000 × g for 20 min to remove pellets; the supernatants contain the crude recombinant protein.

### Protein purification

The recombinant proteins tagged with polyhistidine were purified by Ni-NTA (Nickel-nitrilotriacetic acid) purification system (Invitrogen). All steps were conducted at 4 °C. Lysates were added to prepared purification columns and incubated for 60 min using gentle agitation to keep the resin suspended. The columns were washed with native wash buffer (50 mM NaH2PO4 · H2O, 500 mM NaCl, 20 mM Imidazole, pH 8.0) three times, and were eluted with elution buffer containing increasing concentrations of imidazole (Elution buffer: 50 mM NaH2PO4 · H2O, 500 mM NaCl, 250 mM Imidazole, pH 8.0). Purified proteins were stored at 4 °C for glutaminase activity assay and western blotting. A protease inhibitor cocktail included in the Novagen BugBuster Protein Extraction kit (Millipore, Billerica, MA) was used to prevent proteolysis.

### Western blotting

Protein concentrations of the recombinant protein were determined by BCA Protein Assay Kit (Pierce). Proteins (30 μg) from lysates were electrophoresed on pre-cast SDS-PAGE (sodium dodecyl sulfate-polyacrylamide gel electrophoresis) gels (Bio-Rad, Hercules, CA). Protein separation was performed in gradient gel containing 10% upper gel and 15% lower gel. After electrophoretic transfer to polyvinyldifluoridene (PVDF) membranes (Millipore and Bio-Rad), proteins on the membrane were incubated with polyclonal purified primary antibodies for GAC and KGA (dilution 1:1000, Dr. N. Curthoys, Colorado State University), overnight at 4 °C. The next day, added horseradish peroxidase-linked secondary anti-rabbit (1:10000 dilution, Cell Signaling Technologies, Beverly, MA) antibody was added and the membrane was incubated on a shaker for 1 h at room temperature. Antigen-antibody complexes were visualized by Pierce ECL Western Blotting Substrate (Thermo Fisher Scientific, Waltham, MA).

### Glutaminase activity assay

Glutaminase enzyme activity of recombinant human glutaminase was assessed by glutaminase activity assay using a two-step assay [36, 37]. Briefly, protein concentrations in the lysates were tested using BCA Protein Assay Kit (Pierce). In the first step, fifty milligrams of diluted glutaminase was added to 100 μL of initial assay mix, the mix contained 50 mmol/L glutamine, 0.15 mol/L phosphate, 0.2 mmol/L EDTA, and 50 mmol/L Tris-acetate, pH 8.6, incubated at 37 °C for 30 min. 10 μL of 3 N hydrochloric acid (HCl) was added to inactivate the glutaminase activity and stop the reaction. In the second step, 1 mL of the second reaction mixture was added, which contained 0.4 mg of purified bovine liver glutamate dehydrogenase (Sigma-Aldrich, St. Louis, MO, USA), 0.08 mol/L Tris-acetate, pH 9.4, 0.2 mol/L hydrazine, 0.25 mmol/L ADP (adenosine 5'-diphosphate sodium salt), and 2 mmol/L NAD (β-nicotinamide adenine dinucleotide hydrate). The samples were mixed and incubated for 30 min at room temperature. 100 μL of reaction was used for measurement, the absorbance at 340 nm, and glutamate concentration was determined using a standard curve of 20, 10, 5, 2.5, 1.25, 0.625, and 0.0 mmol/L glutamate, along with negative controls.

### Analysis of extracellular glutamate by RP-HPLC

Glutamate levels were analyzed by reversed phase HPLC (RP-HPLC) using an Agilent 1200 liquid chromatograph and fluorescence detector as previously described [14] with a few modifications. The experiments utilized 4.6 × 75 mm, 3.5 μm ZORBAX Eclipse AAA analytical columns (Agilent). A gradient elution program was optimized for glutamate measurement with a flow rate 0.75 ml/min.

### Statistical analysis

Data are expressed as means ± SD unless otherwise specified. Statistical analysis was performed using ANOVA, followed by the Tukey-post-test for paired observations. Significance was determined by a p value < 0.05. Assays were performed at least three times in triplicate or quadruplicate within each assay.

## Results

### Full-length GAC and KGA have similar enzyme efficacies and kinetics in the glutaminase activity assay

**Fig. 1:**
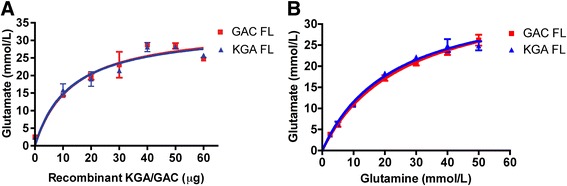
Saturation curve and enzyme kinetics of GAC and KGA. **a**) Recombinant full-length GAC (red) and KGA (blue) at the indicated amount was tested in the glutaminase enzyme activity assay. Glutamate generated in 30 min reaction time was plotted using non-linear regression with GraphPad Prism software. **b**) Enzymatic activity of GAC and KGA was determined by the enzyme activity assay with varying glutamine concentrations. Data was plotted as mmol/L per 30 min reaction time against the glutamine concentration. Saturation profiles represent the non-linear least-squares fit to the Michaelis–Menten equation. Error bars represent the SD for triplicate enzyme activity. FL, full-length

KGA and GAC share the same N-terminal coding sequence but each has its unique C terminal. We have previously demonstrated that GAC, but not KGA, levels were significantly higher in the post mortem brain tissues of HIV dementia individuals than HIV serum negative control [18]. However, it remains unclear whether GAC and KGA have similar efficacies to catalyze glutamine hydrolysis. Using a prokaryotic expression system, we successfully expressed and purified full-length human GAC and KGA proteins. The purified GAC and KGA proteins were tested for their glutaminase activity in a standard glutaminase enzyme activity assay that was described previously [37]. We first studied the enzyme saturated reactions with increasing concentrations of GAC and KGA proteins. Both GAC and KGA generated almost identical dose-dependent increase of glutamate and saturation curve in the glutaminase enzyme activity assay that were supplied with 50 mM glutamine as the substrates, suggesting a close efficacy to produce glutamate for both enzymes (Fig. 1a). To determine the kinetics of enzyme activity, we chose the GAC and KGA concentration that did not generate a saturated reaction in Fig. 1a and subjected them to the enzyme activity assay with varying glutamine concentrations (Fig. 1b). The kinetic analysis revealed that the activities of both full length proteins fit Michaelis–Menten' kinetics model, which is one of the best-known models for enzyme kinetics [38]. GAC and KGA had similar Km for glutamine in the enzyme activity assay (Table 2), consistent with previous studies [39]. Both full-length protein reached similar maximal velocity for the enzyme reaction, confirming that both the enzymes have similar efficacies and kinetics to catalyze glutamine hydrolysis.

**Table 2:** Kinetics of full-length GAC/KGA activity

Protein	Km(mM)	Vmax (μmol/min per ml)
GAC FL	26.02 ± 3.26	39.20 ± 2.26
KGA FL	22.99 ± 3.15	38.04 ± 2.29

### Serial deletions reveal essential N-terminal GAC and KGA AA sequence for glutaminase activity

**Fig. 2:**
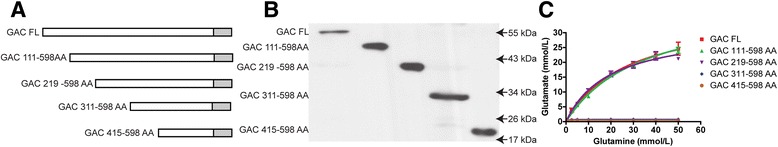
Serial N-terminal deletions of GAC protein. **a**) Schematic illustration of serial N-terminal deletions of GAC was shown. **b**) GAC N-terminal deletion mutant proteins were lysed and subjected to Western blotting with a specific GAC antibody for GAC detection. **c**) Enzymatic activity of each of the mutant was determined by the enzyme activity assay with varying glutamine concentrations. Data was plotted as mmol/L per 30 min reaction time against the glutamine concentration. Non-linear regression was performed with GraphPad Prism software. Results shown are the means ± SD of triplicate samples

**Fig. 3:**
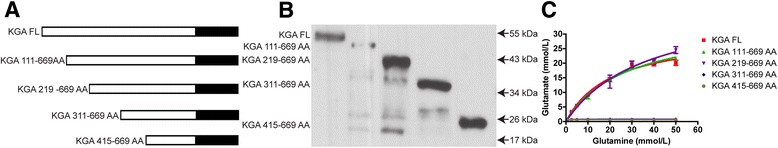
Serial N-terminal deletions of KGA protein. **a**) Schematic illustration of serial N-terminal deletions of KGA was shown. **b**) KGA N-terminal deletion mutant proteins were lysed and subjected to Western blotting with a specific KGA antibody for KGA detection. **c**) Glutaminase activity of each of the mutant was determined by the enzyme activity assay with varying glutamine concentrations. Data was plotted as mmol/L per 30 min reaction time against the glutamine concentration. Non-linear regression was performed with GraphPad Prism software. Results shown are the means ± SD of triplicate samples

To further determine how the N-terminals of the enzymes affect the activity of GAC and KGA, we first performed serial N-terminal deletions of GAC and KGA. Specific primers (Table 1) were designed to generate GAC and KGA mutants with serial N-terminal deletions (Fig. 2a). These mutants were cloned into the pET-28a vector for protein expression. The mutant GAC proteins could be detected by a GAC-specific antibody in Western blotting [40]. The proteins appeared with various sizes in the blot that matched their predicted molecular weight (Fig. 2b). When the first 110 AAs or 218 AAs from N-terminal of GAC were deleted, the truncated GAC retained full glutaminase enzyme activity. In contrast, further removal of the 310 AAs or 414 AAs in N-terminal abrogated glutaminase activity, suggesting that at least part of 218 AA - 310 AA is essential for GAC activity (Fig. 2c, Table 3). Similar to GAC, the mutant KGA proteins could be detected by a KGA-specific antibody [40] (Fig. 3a, b). When the first 110 AAs or 218 AAs from the N-terminal of KGA were deleted, the truncated KGA retained full glutaminase enzyme activity. In contrast, further removal of the 310 AAs or 414 AAs in the N-terminal abrogated glutaminase activity, suggesting that at least part of 218 AA - 310 AA is essential for full KGA activity (Fig. 3b, c) (Table 4).

**Table 3:** Kinetics of the enzyme activity in full-length GAC and its N-terminal deletion mutants

Protein	Km (mM)	Vmax (μmol/min per ml)
GAC FL	27.44 ± 4.92	37.65 ± 3.18
GAC 111–598 AA	34.30 ± 3.88	41.24 ± 2.4
GAC 219–598 AA	19.77 ± 3.17	3 1.61 ± 2.14
GAC 311–598 AA	0.51 ± 0.29	0.78 ± 0.03
GAC 415–598 AA	1.00e-007 ± 0.19	0.52 ± 0.02

**Table 4:** Kinetics of the enzyme activity in full-length KGA and its N-terminal deletion mutants

Protein	Km (mM)	Vmax (μmol/min per ml)
KGA FL	21.23 ± 3.57	30.50 ± 2.18
KGA 111–669 AA	27.37 ± 2.80	34.15 ± 1.64
KGA 219–669 AA	36.61 ± 8.70	41.39 ± 5.19
KGA 311–669 AA	0.43 ± 0.28	0.82 ± 0.03
KGA 415–669 AA	1.00e-007 ± 0.21	0.65 ± 0.02

### Serial deletions reveal essential C-terminal GAC and KGA AA sequence for glutaminase activity

**Fig. 4:**
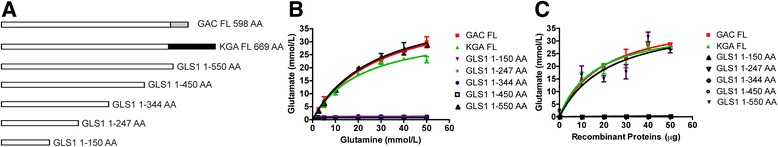
Serial C-terminal deletions of both GAC and KGA proteins. **a**) Schematic illustration of serial C-terminal deletions of GAC and KGA was shown. **b**) Glutaminase activity of each of the mutant was determined by the enzyme activity assay with varying glutamine concentrations. **c**) C-terminal deletions of GAC and KGA were tested in saturation curve of the enzyme activity assay with a fixed glutamine concentration (50 mM). Data was plotted using non-linear regression with GraphPad Prism software. Results shown are the means ± SD of triplicate samples

Next, we performed serial C-terminal deletions of GAC and KGA. GAC and KGA are known to share the same sequence of the 1 AA - 550 AA, while containing different carboxy-terminus. Therefore, when 551 AA - 598 AA were deleted from GAC or 551 AA - 669 AA were deleted from KGA, the truncated proteins are identical (Fig. 4a) and were thus labeled as GLS1. The unique C-terminals of GAC and KGA appeared to bear the antigenic motif recognized by the GAC- and KGA- specific antibodies that were previously described [40, 41]. Consequently, all of the C-terminal deletion mutants for GAC and KGA could not be detected by the antibodies in the Western blots (Data not shown). Through the glutaminase activity assay, we determined that deletion mutants, in which the unique portions of GAC and KGA beyond 550 AA were removed, retained full enzyme activity (Fig. 4b) (Table 5). In contrast, further deletion of 100 AAs from C-terminal resulted in complete loss of enzyme activity for GAC and KGA, suggesting that at least part of 450 AA - 550 AA is essential for full GAC and KGA activity (Fig. 4b) (Table 5). To rule out the possibility that those C-terminal deletion mutants were insufficiently added to the enzyme activity assay, we performed saturation curve of the mutant along with the full-length proteins. Consistent with the data on Fig. 4b, both full-length GAC, KGA, and GLS1 1-550 generated a saturation curve, whereas the remaining mutants failed to do so(Fig. 4c), suggesting that those deletion mutants that had more than100 AAs removed from GAC and KGA C-terminal indeed had no enzyme activities.

**Table 5:** Kinetics of the enzyme activity in full-length GAC, KGA, and their C-terminal deletion mutants

Protein	Km (mM)	Vmax (μmol/min per ml)
GAC FL	28.62 ± 5.37	45.37 ± 4.08
KGA FL	21.69 ± 3.55	35.44 ± 2.48
GLS1 1–150 AA	0.63 ± 0.32	1.30 ± 0.05
GLS1 1 - 247AA	0.69 ± .039	0.92 ± 0.05
GLS1 1–344 AA	0.44 ± 0.26	0.84 ± 0.04
GLS1 1–450 AA	0.66 ± 0.39	0.76 ± 0.03
GLS1 1–550 AA	28.13 ± 3.30	46.14 ± 2.60

### Dual N- and C-terminal deletion of GAC and KGA reveals the core glutaminase catalytic domain

**Fig. 5:**
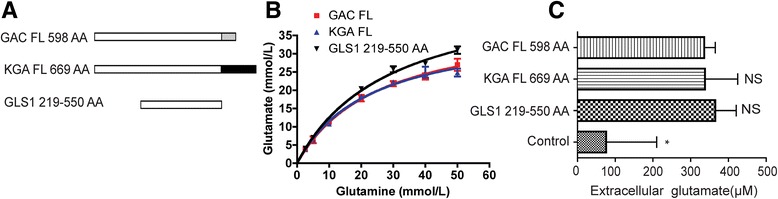
Dual N- and C-terminal deletion of GAC and KGA. **a**) Schematic illustration of the dual N- and C-terminal deletion on GAC and KGA was shown. **b**) Glutaminase activity of each of the mutant was determined by the enzyme activity assay with varying glutamine concentrations. Data was plotted as mmol/L per 30 min reaction time against the glutamine concentration. Non-linear regression was performed with GraphPad Prism software. Results shown are the means ± SD of triplicate samples. **c**) Full-length GAC/KGA, as well as the truncated 219–550 AA GAC/KGA protein were expressed in BL21 cells. The cells were cultured to reach optimal OD values (0.6–0.7) before protein production was induced by IPTG. Glutamate levels in the extracellular fluid were determined by RP-HPLC. Protein purification of the crude lysate derived from the empty vector transfected BL21 cells served as the control. Results shown are the means ± SD of triplicate samples. FL, full-length; NS, non-significant; *, p < 0.05, compared with the activities of FL GAC and KGA

Consistent with N- and C-terminal deletions, when both N- and C-terminal of the KGA and GAC were removed, creating a truncated protein that expressed the central 219 AA - 550 AA (Fig. 5a), the protein retained enzyme activity, suggesting the core enzyme activity of the protein lies within the central 219 AA - 550 AA sequence (Fig. 5b) (Table 6). To further demonstrate the enzyme activity of these mutants in cultured cells, we expressed full length GAC/KGA, as well as the truncated 219 AA - 550 AA coding sequence in BL21 cells. The cells were cultured to reach optimal OD values (0.6–0.7) before protein production was induced by IPTG. The enzyme activity of the protein in the cells was determined by measuring glutamate, which is the product of the enzyme reaction, in the culture supernatant through RP-HPLC. Similar to the enzyme activity data on Fig. 5b, the central 219 AA - 550 AA expression in cells produced glutamate in the extracellular fluid that were comparable to the amount of glutamate produced by full-length KGA and GAC expressions (Fig. 5c). The result suggests that the core 219 AA - 550 AA coding sequence expression in cells maintains the glutaminase enzyme activity.

**Table 6:** Kinetics of the enzyme activity in full-length GAC, KGA, and the dual N- and C-terminal deletion mutant

Protein	Km (mM)	Vmax (μmol/min per ml)
GAC FL	25.43 ± 3.82	40.01 ± 2.74
KGA FL	22.99 ± 3.15	38.04 ± 2.29
GLS1 219–550 AA	29.04 ± 2.9	48.77 ± 2.35

### N- and C-terminal coding regions are essential for the long-term enzymatic activity of GAC and KGA

**Fig. 6:**
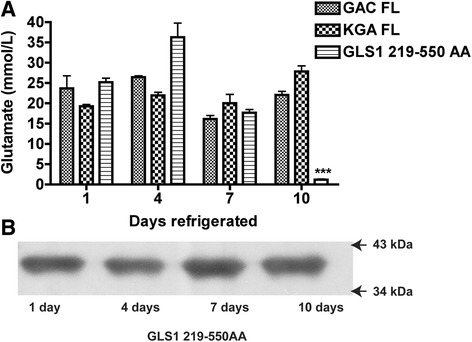
Stability of enzyme activity for the dual N- and C-terminal deletion mutant and full-length GAC and KGA. **a**) Dual N- and C-terminal deletion mutant and full-length GAC and KGA were kept at 4 °C and glutaminase was determined every three days by the enzyme activity assay. **b**) Dual N- and C-terminal deletion mutant were lysed and subjected to Western blotting with a specific His-Tag antibody protein detection

Both 219 AA - 550 AA mutant and full-length GAC and KGA maintained enzymatic activities when kept at 4 °C. Interestingly, when the enzyme activity of these proteins were tested for up to 10 days, the 219 AA - 550 AA truncated protein lost glutaminase activities more readily compared with full-length KGA and GAC, suggesting that the N-terminal and C-terminal coding regions are required for the stability KGA and GAC. (Fig. 6a). To determine whether the loss of glutaminase activity in the dual N- and C-terminal deletion mutant was due to protein degradation, we performed Western blot using a specific His-Tag antibody for the protein amount. His-tagged protein maintained their size and protein amount, suggesting the loss of glutaminase activity in the dual N- and C-terminal deletion mutant was not due to protein degradation.

## Discussion

Glutaminase 1 is a metabolic enzyme important for understanding neurobiology and cancer cells. Although the crystal structures of both full-length protein and the catalytic domain of human glutaminase 1 have been published [32–35], how different coding regions affect the glutaminase enzyme activities remains unclear. In the current study, we successfully cloned human KGA and GAC and subsequently determined that they have similar efficacy and kinetics to catalyze the hydrolysis of glutamine. Furthermore, we used serial deletion and identified that the core enzyme activity of the glutaminase 1 protein is within the central 219 AA - 550 AA. Although N-terminal and C-terminal coding regions outside of the 219 AA - 550 AA appear to be non-essential for the enzyme activity, they are critical for maintaining the long-term activities of the enzymes.

The findings of the structural basis and stability of glutaminase activity are relevant to a number of neurodegerative diseases. In the central nervous system, glutaminase 1 in neurons generates glutamate as an excitatory neurotransmitter. During pathological events, excess glutamate generated by upregulated glutaminase 1 in activated macrophages and microglia plays a key role in inflammation and neurological disorders such as HIV1-associated dementia [18, 24, 25, 37, 42, 43] and multiple sclerosis [44]. We have recently generated a GAC transgenic mouse model, which has GAC overexpression in the brain, to demonstrate a causal association between glutaminase and neurodegenerative diseases (Wang et al. Manuscript in revision). However, data within the current manuscript point to new research directions that warrant further study of the structure and enzyme stability of GAC and KGA.

In cancer cells, glutaminase is an attractive therapeutic target to regulate proliferation and oncogenic transformation [7]. KGA and GAC are typically over-expressed in cancer cells. Interestingly, for breast, lung and prostate tumor cell lines, only the GAC isoform is found within mitochondria [33]. Because of GAC’s exclusive location and efficacious properties, GAC has been suggested to be the key enzyme in mitochondria metabolism in cancer cells [6, 33, 45]. The different expression pattern of GAC and KGA indicates potential functional differences related to the structure of these protein isoforms. In the current studies, we generated the full-length recombinant protein of KGA and GAC. KGA and GAC performed similarly in the enzyme activity saturation studies, suggesting that they have the same efficacy for glutamate generation. Therefore, we found no evidence of specific KGA or GAC function in producing glutamate with the full-length recombinant proteins.

Through the serial deletion approach, we first used Western blot to demonstrate that the truncated proteins had the sizes that were consistent with their predicted molecular weights. However, the Western blot was not intended to precisely quantify protein amount due to several factors, including protein expression efficiency, degradation, extraction method, and antibody binding affinity, affect the quantification in Western blot. In the subsequent enzyme activity assays, all proteins were quantified through a Bradford protein assay and the same protein concentrations were used. Through the enzyme activity assay, we have demonstrated that the core enzyme activity of the KGA and GAC proteins lies within the central 219 AA - 550 AA. The serial deletion approach allows us to understand the structural basis of GLS1 action. Indeed, when 110 AAs or 218 AAs were deleted from the N-terminal, KGA and GAC retained similar enzyme activity of the full length proteins. In contrast, deletion of 310 AAs resulted in a complete loss of enzyme activity for KGA and GAC, suggesting that at least part of 218 AA - 310 AA is essential for full KGA and GAC activity. A similar analysis at the C-terminal has concluded that at least part of 450 AA - 550 AA is essential for full KGA and GAC activity. We have not identified the exact AA in 218 AA - 310 AA and 450 AA - 550 AA that is required for the core glutaminase enzyme activity. These experiments remain a target for further investigation.

We also analyzed the truncated glutaminase protein with the central 219 AA - 550 AA. The protein retained high glutaminase activity, suggesting that the section 219 AA - 550 AA is the main catalytic domain for KGA and GAC. This result is consistent with the crystal structures of KGA with 221 AA - 533 AA as catalytic domain [34]. Taken together, we have determined that human KGA and GAC have similar kinetics and efficiency to hydrolyze glutamine into glutamate. Serial deletion analysis of human KGA and GAC recombinant protein reveals a core enzyme activity domain that spans 219 AA - 550 AA. The novel discovery of the coding sequence for the core glutaminase function, as well as the finding that.

N-terminal and C-terminal coding regions outside of the 219 AA - 550 AA that are critical for the long-term activities of the enzymes, provides insight to the structural basis of GLS1 activity.

## Conclusions

In conclusion, we have determined that both KGA and GAC isoforms have similar efficacy and kinetics to catalyze the hydrolysis of glutamine. Although N-terminal and C-terminal coding regions outside the core catalytic domain of GAC and KGA appear to be non-essential, they are critical for the long-term activities of the enzymes. This study provides important knowledge to understand the structural basis of the glutaminase 1 enzyme. Also, understanding the function of KGA and GAC in both physiological and pathological systems as it relates to neurodegeneration can be used for drug development that target glutaminase 1 enzyme for therapeutic purposes in cancer and neuroscience.

## Abbreviation

AA: Amino acid
ADP: Adenosine 5'-diphosphate sodium salt
EDTA: Ethylenediaminetetraacetic acid
FL: Full length
GAC: Glutaminase C
GLS: Glutaminase
IPTG: Isopropyl β-D-1-thiogalactopy-ranoside
KGA: Kidney glutaminase
LB: Luria broth
NAD: β-nicotinamide adenine dinucleotide hydrate
Ni-NTA: Nickel-nitrilotriacetic acid
PVDF: Polyvinyldifluoridene.
